# Effects of Mycotoxins on the Intestine

**DOI:** 10.3390/toxins11030159

**Published:** 2019-03-13

**Authors:** Imourana Alassane-Kpembi, Philippe Pinton, Isabelle P. Oswald

**Affiliations:** 1 Toxalim (Research Centre in Food Toxicology), Université de Toulouse, INRA, ENVT, INP-Purpan, UPS, 31027 Toulouse, France; imourana.alassane-kpembi@inra.fr (I.A.-K.); philippe.pinton@inra.fr (P.P.); 2 Ecole Polytechnique d’Abomey-Calavi, Université d’Abomey-Calavi, 01BP2009 Abomey-Calavi, Bénin

The gastrointestinal tract is the first physiological barrier against food contaminants, as well as the first target for these toxicants. As prominent food and feed contaminants, mycotoxins frequently come into contact with the intestinal mucosa, and awareness of their potentially deleterious effects is increasing [1,2]. Even though the mucosa is a major functional element of intestinal integrity, increasing evidence suggests that other constituents, such as mucus and microbiota, are also involved [3]. This special issue reports on recent progress in the characterization of the intestinal toxicity of mycotoxins. 

Substantial data have been assembled on the damage caused by mycotoxins to a number of histological structures and functions of the intestinal tissue. Mycotoxins, with chemical structures as diverse as aflatoxins, ochratoxin, and deoxynivalenol (DON), have been shown to impair intestinal permeability in species as different as humans, fish, and pigs, removing any remaining doubt about global mycotoxin-driven alteration of the intestinal barrier function [4,5,6]. The mucus and its goblet cell producers are underestimated players that have long escaped the attention of the mycotoxicology community when assessing the barrier function [3,7]. A light and electron microscopy study by Przybylska-Gornowicz et al. [8] investigated the fate of goblet cells and their mucus production in a pig colon exposed to the *Fusarium* toxins, DON and zearalenone (ZEN), at supposedly non-toxic levels. 

Enteric neurons involved in many regulatory processes, connected with all aspects of intestinal physiology, have also been underestimated, and the question of whether mycotoxins could target the enteric nervous system (ENS) deserves attention. Makowska et al. [9] demonstrated that following the exposure of pigs to low doses of the T-2 toxin, even the ENS undergoes adaptive and reparative processes, possibly resulting in changes in the chemical coding of the neurons and nerve fibers in the porcine stomach and duodenum. 

An overview of the detrimental effects of mycotoxins on the intestine could not ignore the gut-hosted microbiota that are now regarded as a fully fledged organ associated with the gut [10]. Yang et al. [11] reported dramatic changes in mouse-digestive microbiota, following long-term feeding with aflatoxin B_1_. Reddy et al. [12] analyzed the colon content of pigs fed with DON or ZEN and reported that both mycotoxins favored the abundance of the *Lactobacillus* genus, suggesting that members of this genus could play a key role in the detoxification of dietary DON and ZEN in pigs. Also in pigs, dietary fumonisin B_1_ (FB_1_) was shown to hinder the age-related dynamic of fecal microbiota, starting from 15 days of exposure [13]. 

The emergence of the intestine as a critical target for mycotoxin toxicity concurrently raises the question of the suitability of current regulations to protect against alterations of this organ. Maruo et al. [14] concluded that ergot alkaloids that contaminate feed, but at rates under the current EU regulatory limits, still damage the intestine. Likewise, Cieplinska et al. [15] reported that the cecal water obtained from pigs fed ZEN at no-observed-adverse-effect-level (NOAEL) and below, still had a significant genotoxic effect, highlighting the need for further investigation into the specific sensitivity of the intestine to mycotoxins. 

Finally, the unavoidable presence of mycotoxins in animal feed, despite continuing efforts to keep the risk under control, calls for the implementation of new detoxification strategies, whose efficacy still needs to be assessed [16]. To that end, the intestinal toxicity of mycotoxins offers several possibilities. Alassane-Kpembi et al. [17] performed a whole-transcriptome analysis to decipher the early response of the small intestine to the deleterious effects of DON after administration of the *Saccharomyces cerevisiae* boulardii strain CNCM I-1079. These authors reported that applying the yeast significantly reduced the overall impact of DON on the transcriptome, and specifically reversed a number of signaling pathways triggering inflammation, oxidative stress, and lipid metabolism. Likewise, the oxidative stress and mitochondrial apoptosis induced by ZEN in pig intestinal epithelial cells were reported to be alleviated by application of N-Acetylcysteine [18]. Dietary supplementation with the *Clostridium* sp. WJ06 strain as a DON detoxification strategy in pigs also appears to be of potential interest, as Li et al. [19] showed that this bacterial strain significantly attenuated the toxicity of DON, while simultaneously modulating the intestinal micro-ecosystem of growing pigs. Hypothesizing that the toxicity of mycotoxins can be counteracted through specific adjustments of the composition of intestinal microflora, Zheng et al. [20] explored the effects of administering hydrogen-rich water and lactulose, two hydrogen-producing prebiotics, on the microbiota imbalance induced by *Fusarium* mycotoxins in piglets. These authors showed that providing functional hydrogen to the pig gut could protect the animal against the imbalance of intestinal communities of microbiota, and protect it from a reduction in the production of short-chain fatty acids and a higher rate of diarrhea induced by a mix of *Fusarium* mycotoxins. Conversely, despite their broadly acknowledged gut health promoting action, chito-oligosaccharides had no remediating effect against the intestinal toxicity of DON [21].

This special issue contains original contributions that advance our knowledge of the intestinal toxicity of mycotoxins. Most of the studies focus on fusariotoxins, but the toxicity of aflatoxins and ergot alkaloids is also addressed. Mycotoxin toxicity is investigated on different cellular targets (epithelial cells, goblet cells, and neurons), markers (oxidative stress, permeability), and the intestinal bacterial flora. The use of the pig model was recurrent in in vivo studies, making it possible to envisage dual valorization of the present findings in biomedical and agricultural research. An original contribution on salmon provides useful information for this breeding species, which remains poorly investigated in the field of mycotoxicology. The outcomes of this special issue improve the characterization of the deleterious effects of mycotoxins on the intestine and identify potential solutions to mitigate these effects. The different detoxification strategies described here will certainly attract the attention of the scientific community.
